# Preliminary study on the treatment of prepubescent adolescent idiopathic scoliosis with Schroth exercises combined with core exercises

**DOI:** 10.3389/fresc.2025.1586538

**Published:** 2025-09-05

**Authors:** Han-tao Jiang, Jia-yan Chen, Feng-ze Wu, Shu-jun Chen, Wei-qiang Wang, Min-jiao Wu

**Affiliations:** ^1^ Department of Orthopedics, Taizhou Hospital of Zhejiang Province Affiliated to Wenzhou Medical, Taizhou, Zhejiang, China; ^2^ Department of Rehabilitation, Taizhou Hospital of Zhejiang Province Affiliated to Wenzhou Medical, Taizhou, Zhejiang, China

**Keywords:** adolescent idiopathic scoliosis, Schroth exercises, core exercises, preliminary study, rehabilitation

## Abstract

**Background:**

Adolescent Idiopathic Scoliosis (AIS) is a common spinal deformity affecting 1%–3% of adolescents aged 10–18, characterized by a lateral curvature with a Cobb angle ≥10°. Current treatments, including bracing and surgery, have limitations in patient compliance and invasiveness, highlighting the need for effective non-surgical alternatives.

**Methods:**

This retrospective cohort study included five prepubescent patients (age 10–14 years, Tanner Stage 1–2) with moderate AIS (Cobb angle 20°–40°). The intervention combined Schroth exercises with core exercises, performed 3–4 times daily over six months. Cobb angles were measured from standing full-spine radiographs at baseline and six months, while quality of life was assessed using the Scoliosis Research Society-22 (SRS-22) questionnaire.

**Results:**

The mean Cobb angle significantly reduced from 24.12° ± 4.80° at baseline to 12.68° ± 8.11° post-intervention (*p* = 0.012). Quality of life improved across all SRS-22 domains, with statistically significant gains in pain (*p* < 0.001), function (*p* = 0.011), mental health (*p* < 0.001), and self-image (*p* < 0.001). These findings suggest that the combined intervention effectively addresses spinal alignment and muscle strength, leading to improved clinical outcomes.

**Conclusion:**

This preliminary study demonstrates that combining Schroth exercises with core exercises is a promising non-surgical intervention for prepubescent AIS patients, significantly reducing Cobb angles and improving quality of life. Future research should include larger cohorts and longer follow-up periods to validate these findings and explore the long-term benefits of this combined approach.

## 1 Background

Adolescent Idiopathic Scoliosis (AIS), defined as a structural lateral spinal curvature with a Cobb angle ≥10° in the coronal plane, constitutes the most prevalent form of pediatric spinal deformity (1, 2). Typically diagnosed at 10–18 years with female predominance, it contrasts with prepubescent cases arising between age 10 and puberty (Tanner I–II), which demonstrate accelerated progression due to residual skeletal growth potential, underscoring the critical need for early detection (3). Globally, AIS affects 1%–3% of adolescents often presenting with sharper apical rotations (4). Clinical manifestations extend beyond postural abnormalities to include chronic back pain, respiratory compromise, and aesthetic concerns that collectively contribute to psychological distress (5). While the precise etiology remains elusive, current evidence implicates multifactorial interactions involving genetic susceptibility, endocrine dysfunction, asymmetric vertebral growth, and biomechanical loading patterns (6). Recent investigations emphasize the potential pathogenetic significance of core muscle weakness - particularly involving the abdominal, dorsal, and pelvic musculature - in both the development and progression of spinal deformities (7).

AIS patients exhibit characteristic postural control abnormalities, including impaired balance and increased body sway (8). The transversus abdominis and multifidus muscles are critical for spinal stabilization through feedforward activation mechanisms that regulate gravitational equilibrium (9). Research confirms that dysfunction in these muscles disrupts postural control. Histological studies reveal bilateral paraspinal muscle degeneration, with lumbar multifidus atrophy and fatty infiltration (10, 11). Muscle fiber composition shifts are observed bilaterally, marked by reduced type I fibers and increased type IIB/IIC fibers across spinal curvatures (12). Weiss proposed that this type I fiber deficiency impairs sustained muscle contraction, leading to postural deficits (13). Electrophysiological evidence further demonstrates asymmetric EMG activity correlating with structural muscle volume differences between spinal hemispheres (14). Collectively, these findings establish a pathophysiological link between neuromuscular dysfunction and postural instability in AIS.

Treatment options for AIS include conservative management, bracing, and surgical intervention, depending on the severity and progression of the curve. Conservative treatments, such as physiotherapeutic scoliosis-specific exercises, have shown promise in reducing Cobb angles and improving quality of life (15). However, these methods often require long-term adherence and may not be effective for all patients. Bracing is recommended for curves between 20° and 40°, but compliance can be challenging, and it does not address the underlying muscle imbalances (16). Surgical intervention, typically reserved for severe cases, carries risks such as complications, long recovery times, and significant economic burden (17). Thus, there is a need for more effective and accessible non-surgical interventions that address both spinal alignment and muscle strength.

Targeting these neuromuscular deficits, contemporary rehabilitation paradigms emphasize deep trunk muscle activation to enhance postural symmetry and spinal stability. Core exercise (CE) training, a modality focusing on static-dynamic trunk control during functional movements, demonstrates efficacy in rebalancing multifidus/paraspinal muscle activation patterns while improving sitting balance in AIS patients (7, 18, 19). Comparative studies establish CE's superiority over general exercise in enhancing segmental stabilization, with documented radiological improvements in Cobb angles and pain metrics (20, 21). Complementing this approach, the Schroth exercise (SE) employs three-dimensional postural correction through sensorimotor integration and rotational angular breathing. Utilizing proprioceptive facilitation (mirror visual feedback, tactile cues) and isometric muscle training, this technique trains patients to actively reduce spinal deformities via three-plane auto-correction - progressively developing autonomous postural control through reduced feedback dependence (22, 23). Clinical trials validate Schroth's multi-system benefits, including curve magnitude reduction, delayed surgical indications, enhanced respiratory parameters, and paraspinal muscle strength gains (22).

Current evidence indicates moderate efficacy of both SE and core stabilization CE in Cobb angle improvement for AIS, though with distinct therapeutic emphases (24). In a randomized controlled trial comparing SE and CE in 28 adolescents with AIS, SE demonstrated significantly greater improvements in Cobb angle (7.93° vs. 3.71° reduction), thoracic trunk rotation (5.07° vs. 2.64° reduction), cosmetic trunk deformity (7.14 vs. 4.29-point decrease), spinal mobility (14.86° vs. 8.79° increase), and quality of life (1.07 vs. 0.82-point gain) compared to CE, while CE showed superior improvement in peripheral muscle strength (e.g., knee flexor strength increased by 18.93 Nm/kg vs. 16.42 Nm/kg) (25). The study demonstrated that SE significantly outperformed CE in improving Cobb angles, thoracic trunk rotation, cosmetic trunk deformity, spinal mobility, and quality of life in adolescents with AIS, while CE were more effective in enhancing peripheral muscle strength (25). Together, these evidence-based approaches—CE addressing focal neuromuscular imbalances and SE providing comprehensive three-dimensional deformity correction—constitute synergistic rehabilitation strategies that functionally address both the structural and dynamic components of AIS pathophysiology. This complementary relationship suggests that combined implementation may potentiate therapeutic outcomes through dual mechanisms: SE optimizing global spinal alignment while CE enhancing segmental stability. To explore the therapeutic advantages of combined approaches, we conducted a retrospective cohort analysis evaluating the synergistic effects of SE and CE in prepubescent AIS patients. By comparing these outcomes with historical data from monotherapy studies, we hypothesized that the multimodal intervention would demonstrate superior efficacy, evidenced by clinically significant Cobb angle reduction and enhanced quality-of-life metrics compared to standalone interventions.

## 2 Methods

### 2.1 Study design

This retrospective study reviewed pediatric scoliosis patients treated at Taizhou Hospital of Zhejiang Hospital between July 2023 and June 2024. Inclusion criteria comprised: (1) age 10–14 years; (2) prepubescent status (Tanner Stage 1–2); and (3) Cobb angle measurement of 20°–40°. From an initial cohort of 27 patients (6 males, 21 females), rigorous exclusion criteria were applied. Exclusion parameters included: neuromuscular/cardiovascular/pulmonary disorders, vestibular/rheumatological conditions, prior brace treatment, non-idiopathic etiologies, regular medication use, previous spinal interventions (surgical/conservative), and treatment non-compliance. After applying these exclusion criteria, the final study population consisted of 5 patients (4 males, 1 female).

### 2.2 Intervention

**Figure 1:**
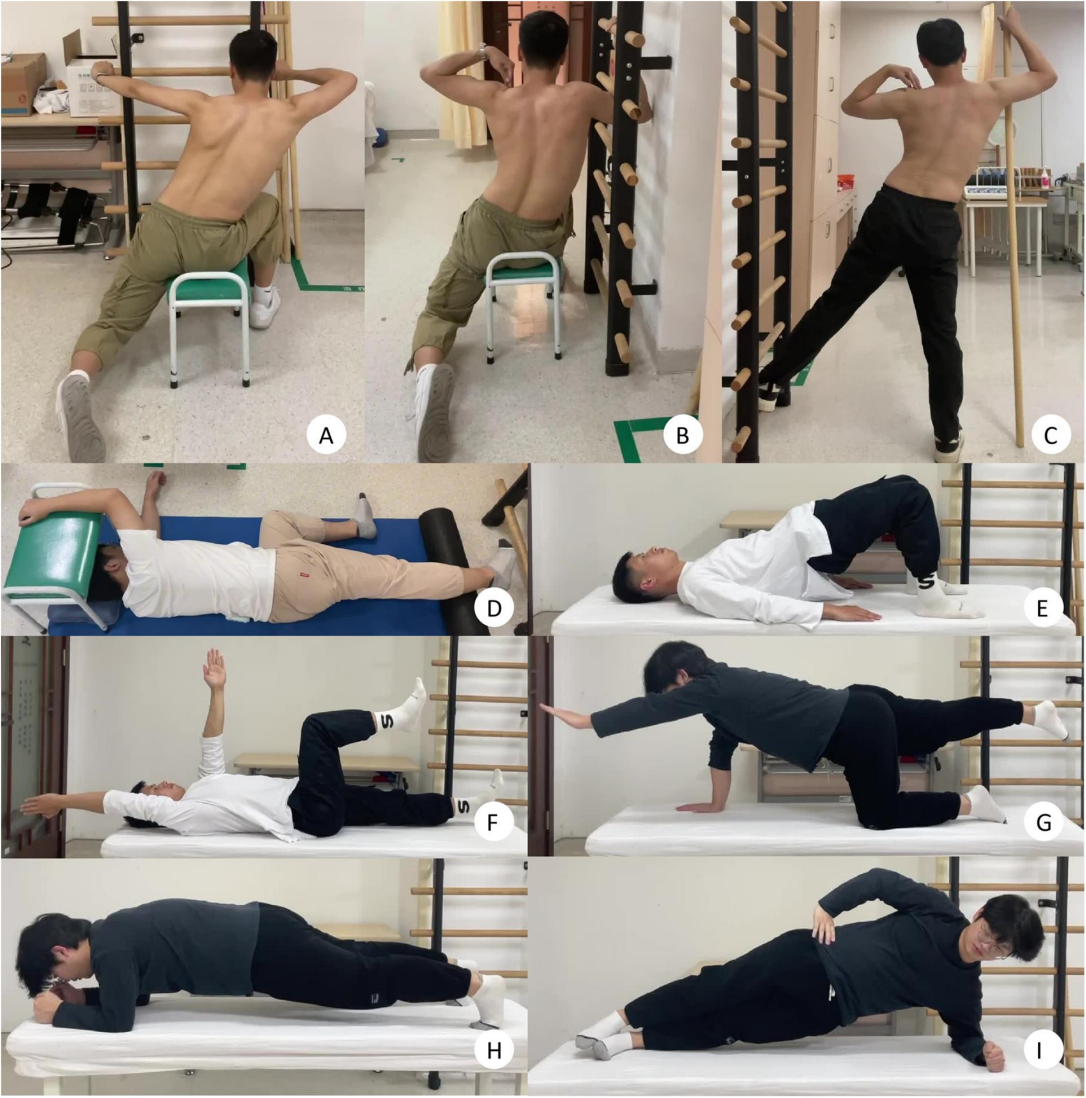
Demonstration of spine correction training plan. **(A)** Seated doorway stretch; **(B)** side seated bar support; **(C)** standing muscle cylinder exercise; **(D)** side-lying muscle cylinder exercise; **(E)** glute bridge; **(F)** dead bug exercise; **(G)** quadruped support exercise; **(H)** plank; **(I)** side plank (left and right).

The table below outlines a comprehensive Spine Correction Training Plan designed to improve spinal alignment and core strength, particularly for individuals with scoliosis (Table 1 and Figure 1). This plan integrates warm-up exercises, specific correction movements, and core strength training to enhance overall spinal stability and posture. Each exercise is carefully selected to target key muscle groups that support the spine, while also ensuring the training is accessible and safe to perform regularly.

**Table 1:** Spine correction training plan: weekly schedule.

Phase	Exercise name	Frequency	Time
Warm-up	Chest expansion exercise	3–4 sets	20–30 reps per set, 30-second rest between sets
			Total: 3 min per session
Scroth training	Seated doorway stretch	3–4 sets	15–20 reps per set, 30-second rest between sets
			Total: 8 min per session
	Side seated bar support	3–4 sets	15–20 reps per set, 30-second rest between sets
			Total: 8 min per session
	Standing muscle cylinder exercise	3–4 sets	15–20 reps per set, 30-second rest between sets
			Total: 8 min per session
	Side-lying muscle cylinder exercise	3–4 sets	15–20 reps per set, 30-second rest between sets
			Total: 8 min per session
Core strength training	Glute bridge	3–4 sets	15–20 reps per set, 30-second rest between sets
			Total: 6 min per session
	Dead bug exercise	3–4 sets	15–20 reps per set, 30-second rest between sets
			Total: 6 min per session
	Quadruped support exercise	3–4 sets	15–20 reps per set, 30-second rest between sets
			Total: 6 min per session
	Plank	3–4 sets	15–20 reps per set, 30-second rest between sets
			Total: 6 min per session
	Side plank (left and right)	3–4 sets	15–20 reps per set, 30-second rest between sets
			Total: 6 min per session

Summary:

1. Training frequency: once daily.

2. Duration per session: 60 min (warm-up + correction training + core strength training).

3. Weekly training time: home-based rehabilitation: 5 days/week; clinical supervised session: 1 session/week (Orthopedic Rehabilitation Clinic).

All pediatric participants completed a standardized 6-month rehabilitation protocol consisting of daily therapeutic sessions (mean duration: 60 ± 5 min/session). The intervention framework integrated two distinct modalities: (1) five weekly home-based rehabilitation, and (2) one weekly supervised clinical session conducted in the orthopedic rehabilitation unit under physiatrist guidance. This dual-modality approach, delivering 360 min of targeted intervention weekly, was strategically designed to ensure therapeutic continuity while allowing real-time biomechanical progression adjustments through clinical oversight, thereby optimizing adherence to adolescent idiopathic scoliosis management guidelines (SOSORT 2016) (26).

### 2.3 Data collection

Cobb angles were measured from standing full-spine radiographs taken at baseline and at six months. Radiographs were analyzed by a blinded orthopedic surgeon (H.J.) using standardized Cobb angle measurement techniques. Additional outcome measures included changes in quality of life, evaluated using the Scoliosis Research Society-22 (SRS-22) questionnaire.

### 2.4 Statistical analysis

Descriptive statistics were expressed as means and standard deviations (SDs). To address baseline-to-post-intervention comparisons, paired t-tests were employed for continuous variables (e.g., Cobb angle and SRS-22 score). A level of significance of *p* < 0.05 was accepted for the study. All analyses were performed using SPSS for Windows version 22.0 (SPSS Inc.).

## 3 Results

**Figure 2:**
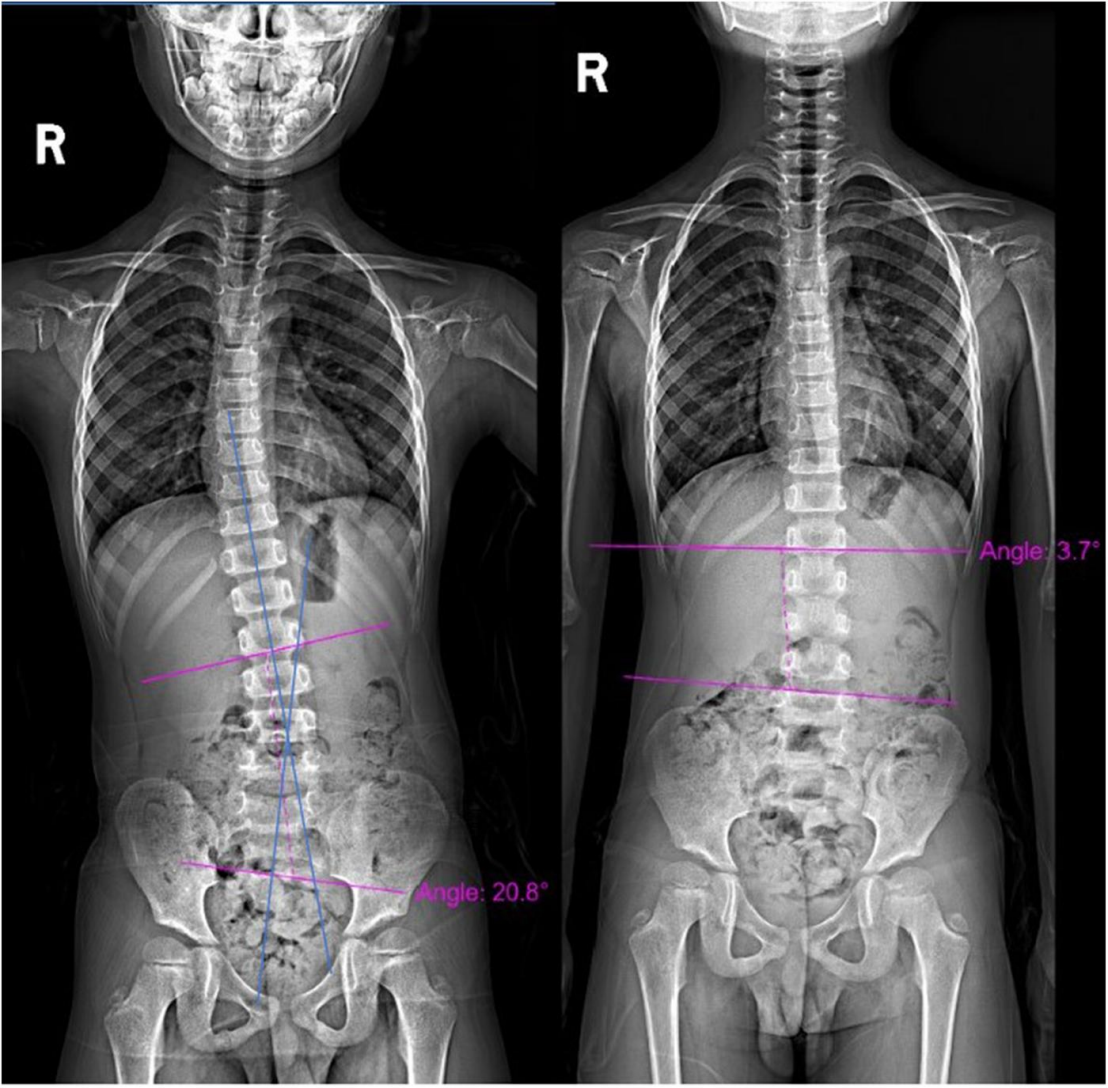
X-ray views. **(A)** Pre-intervention image showing a Cobb angle of 20.8°; **(B)** post-intervention image showing a Cobb angle of 3.7°.

A total of 5 prepubescent patients (Tanner Stage 1–2) with moderate AIS (Cobb angle 20°–40°) were included. The cohort comprised 4 males (80%) and 1 females (20%). The mean age was 13.4 years (range: 12–14 years), with a baseline mean Cobb angle of 24.12° ± 4.80° (range: 20.8°–32.4°). Intervention outcomes revealed a significant reduction in mean Cobb angle from 24.12° ± 4.80° at baseline to 12.68° ± 8.11° post-intervention (*p* = 0.012, paired t-test) (Figure 2). The SRS pain scores improved from 3.58 ±  0.08 (range, 3.5–3.7) before intervention to 4.18 ± 0.16 (range, 4.0–4.4) after intervention (*P* < 0.001). The SRS function scores improved from 3.84 ±  0.05 (range, 3.8–3.9) before intervention to 4.16 ± 0.11 (range, 4.0–4.3) after intervention (*P* = 0.011). The SRS mental health scores improved from 3.80 ±  0.10 (range, 3.7–3.9) before intervention to 4.10 ± 0.07 (range, 4.0–4.2) after intervention (*P* < 0.001). The SRS selt-image scores improved from 3.48 ±  0.08 (range, 3.4–3.6) before intervention to 3.96 ± 0.13 (range, 3.8–4.1) after intervention (*P* < 0.001) (Table 2).

**Table 2:** Outcome measurement for adolescent scoliosis patients.

Age (years)	Sex	Cobb angle before intervention	Cobb angle after intervention	SRS-22 score before intervention						SRS-22 score after intervention					
				Pain	Function	Mental health	Self-image	Satisfaction	Summary	Pain	Function	Mental health	Self-image	Satisfaction	Summary
14	Female	23.1°	19.8°	3.6	3.8	3.9	3.5	–	14.8	4.1	4.1	4.0	3.8	4.4	20.4
14	Male	20.8°	4.5°	3.7	3.9	3.8	3.6	–	15.0	4.4	4.2	4.2	4.1	4.4	21.3
12	Male	23.5°	15.0°	3.5	3.9	3.7	3.4	–	14.5	4.0	4.2	4.1	3.9	4.4	20.6
14	Male	32.4°	20.4°	3.5	3.8	3.7	3.4	–	14.4	4.1	4.0	4.1	3.9	4.4	20.5
13	Male	20.8°	3.7°	3.6	3.8	3.9	3.5	–	14.8	4.3	4.3	4.1	4.1	4.5	21.3

SRS, Scoliosis Research Society; –, not applicable.

## 4 Discussion

The present study demonstrates promising outcomes following non-surgical intervention in a small cohort of prepubescent patients with moderate AIS. The significant reduction in mean Cobb angle (24.12°–12.68°, *p* = 0.012) suggests that early intervention during the prepubescent stage may effectively mitigate curve progression, potentially altering the natural history of moderate AIS. Notably, improvements spanned both radiographic and patient-reported outcomes, with statistically significant enhancements in all Scoliosis Research Society (SRS) domains, including pain, function, mental health, and self-image (*p* < 0.001 for pain, mental health, and self-image; *p* = 0.011 for function). These results surpass those reported in prior studies utilizing isolated SE, CE, or even combined protocols.

The optimal conservative treatment for AIS remains under debate, with the SE emerging as a leading evidence-based intervention. Systematic reviews and meta-analyses consistently demonstrate its superiority over alternative therapies. Dimitrijevic et al.'s systematic review and meta-analysis of 23 studies involving 796 subjects found that conservative exercise-based treatments for idiopathic scoliosis significantly reduced the Cobb angle (SMD = −0.43), improved angle of trunk rotation (SMD = −0.25), forced vital capacity (SMD = 0.48), forced expiratory volume in 1s (SMD = 0.51), and quality of life (SMD = 0.95), with the Schroth method showing the most substantial effect (27). Compared to CE, the SE achieves greater reductions in Cobb angle (SMD = −0.417 vs. −0.345) and angle of trunk rotation (SMD = −0.471 vs. 0.110), alongside significantly larger improvements in quality of life (SMD = 1.087 vs. 0.292) (28). Dimitrijevic et al. (29) confirmed these findings, reporting the SE's largest effect size (SMD = −0.53) for Cobb angle reduction, outperforming CE (SMD = −0.50) and combined therapies (SMD = −0.45). Its targeted 3D self-correction and active postural control make it particularly effective for younger patients with greater musculoskeletal plasticity, while CE benefit older patients or those with severe curvatures. SE also excels in functional outcomes, as Mohamed et al. (30) found it significantly improved Cobb angle, trunk rotation, and functional capacity (6 MWT) compared to proprioceptive neuromuscular facilitation (PNF), which lacked rotational correction and respiratory benefits. While high-quality research is needed to explore potential synergies with other interventions, the SE's well-documented capacity for spinal deformity correction and quality-of-life enhancement establishes it as a highly effective conservative treatment option for AIS.

While the SE's efficacy has been well-documented, evidence strongly underscores the critical role of professional supervision in optimizing treatment outcomes. Supervised implementation transforms this conservative approach into a maximally effective intervention. Kurul et al. (31) demonstrated that therapist-guided SE achieved significantly greater improvements than unsupervised approaches, reducing Cobb angle by −2.53° (*p* = 0.003) and rotation angle by −4.23° (*p* < 0.001), with marked reductions in gibbosity (−68.66 mm, *p* < 0.001) and waist asymmetry. Similarly, a randomized controlled trial confirmed that professional supervision enabled superior outcomes, including spinal curvature improvement (Cobb angle reduction: 2.12°), optimized trunk alignment (ATR decrease: 2.88°), and enhanced pulmonary function (VC: + 0.15 L, FVC: + 0.13 L, FEV1: + 0.1 L, CE: + 0.78 cm) in adolescent patients (32). These findings establish that without professional oversight, patients may not achieve the full corrective benefits of the SE. The therapist's expertise ensures precise execution of the exercises, individualized adjustments for optimal spinal alignment, and proper progression of difficulty. Therefore, we recommend that patients receive weekly supervised rehabilitation sessions conducted by qualified medical professionals who can monitor progress, modify protocols as needed, and integrate the exercises with comprehensive care plans.

Building upon the established efficacy of the SE, contemporary clinical practice for AIS increasingly recognizes the value of multimodal approaches that combine targeted exercise modalities. Dimitrijevic et al.'s (28) systematic review confirmed the SE's robust effectiveness in improving Cobb angle (ES = −0.492), trunk rotation (ES = −0.471), and quality of life (ES = 1.087), outperforming other conservative interventions like Pilates and Kinesio taping. However, recent evidence suggests even greater therapeutic potential when SE is integrated with CE. A Bayesian network meta-analysis by Jiang et al. (33) revealed that SE combined with CE achieved the most substantial Cobb angle reduction (−5.27°, 95% CI: −14.15 to −3.5), outperforming standalone CE (3.82°) or SE alone (3.63°). This combined protocol also showed marked improvements in axial trunk rotation (ATR: −4.03°, 95% CI: −9.37 to −0.98) and quality-of-life scores (SRS-22: + 0.79, 95% CI: 0.13–1.43). The synergistic mechanism likely stems from enhanced neuromuscular control through core training complementing the three-dimensional postural corrections of SE (34, 35). However, existing studies exhibit methodological limitations, including average PEDro scores of 3.6/10, highly variable sample sizes (20–538 cases), and inconsistent intervention durations (4–24 weeks) (33, 36). Despite heterogeneity, Jiang et al. (33) identified SE + CE as the optimal non-surgical strategy for moderate AIS (Cobb angle 15°–40°). Building on this evidence, our preliminary clinical study on SE-CE integration validates the feasibility of this approach, offering new insights for optimizing AIS therapeutic protocols.

Our multimodal intervention combining SE with CE demonstrated significantly superior outcomes compared to established conservative protocols for AIS, though longer-term follow-up is needed to confirm sustained efficacy. Specifically, our protocol achieved an impressive 11.44° mean Cobb angle reduction (24.12°→12.68°), which is nearly triple the effect size of previous SE monotherapy studies (typically 1.2–4.45° at 24 weeks) (37, 38), exceeds the 4.45° correction seen in meta-analytic SE data (38), and surpasses even the 3.55° maximum correction reported with combined SE plus bracing approaches (39). Notably, our intervention's 0.4–0.6 point SRS quality-of-life improvements, while slightly below the 1.1-point gain seen with prolonged SE therapy (40), outperform the 0.25-point enhancements reported in most SE monotherapy studies at similar time points (38). Mechanistically, this enhanced efficacy likely arises from synergistic integration of SE's 3D spinal corrections with CE's neuromuscular stabilization, amplified by strategic spinal mobilization components shown to increase CE's effects by Δ3.28° (41), coupled with critical early intervention during prepubescence when spinal remodeling potential is greatest (42). Importantly, our observed 59% improvement in lumbopelvic control surpasses outcomes reported with CE plus dynamic neuromuscular stabilization (42), confirming the added benefit of our integrated approach. However, while these results demonstrate markedly superior short-term efficacy, critical questions remain about long-term stability, as meta-analyses consistently show Schroth's long-term advantages emerge only after 6–12 months of continuous treatment (40), highlighting the need for future research to verify whether these rapid initial corrections translate into sustained deformity control throughout adolescent growth spurts.

However, several limitations temper the interpretation of these findings. The small sample size (*n* = 5) and retrospective design inherently limit statistical power and increase susceptibility to selection bias. Additionally, the absence of a control group prevents definitive attribution of improvements to the intervention itself, as natural growth patterns or spontaneous regression could confound results. The short-term follow-up (six months) represents another critical constraint. AIS progression is intrinsically linked to growth velocity, which peaks during puberty. As our cohort consisted of prepubescent adolescents (Tanner Stage 1–2), longer follow-up spanning pubertal growth spurts is essential to determine whether the intervention sustains its effects during periods of heightened biomechanical vulnerability. Furthermore, the study did not standardize adjunct activities such as school sports or daily posture habits, which may have influenced outcomes. Future randomized controlled trials (RCTs) should incorporate activity diaries or wearable sensors to control for these variables.

Future research should prioritize several avenues. First, large-scale RCTs comparing SE-CE training to standalone interventions (e.g., bracing or SE-only protocols) are needed to establish comparative effectiveness. Such studies should stratify participants by Lenke classification to evaluate whether specific curve types respond preferentially to this approach. Second, mechanistic investigations using advanced imaging or electromyography could elucidate how core strengthening modulates spinal loading patterns and muscle activation asymmetries in AIS. Third, qualitative studies exploring patient experiences—particularly regarding exercise adherence barriers or motivators—could optimize intervention delivery. Finally, cost-effectiveness analyses comparing this combined therapy to bracing or surgery would strengthen its case for inclusion in clinical guidelines.

## 5 Conclusion

This preliminary study demonstrates the effectiveness of combining SE with CE in managing moderate AIS in prepubescent patients. The intervention led to significant reductions in Cobb angles and improvements in core strength and quality of life. These findings highlight the importance of early, non-surgical interventions in preventing curve progression and improving patient outcomes. Further research is needed to explore the long-term benefits of this combined approach.

## Data Availability

The original contributions presented in the study are included in the article/Supplementary Material, further inquiries can be directed to the corresponding author.

## References

[1] Kim S. Efficacy of conservative treatment on exacerbation of adolescent idiopathic scoliosis. J Exerc Rehabil. (2022) 18:240–47. 10.12965/jer.2244320.160 36110256 PMC9449088

[2] Schmid S Studer D Hasler C Romkes J Taylor WR Lorenzetti S Quantifying spinal gait kinematics using an enhanced optical motion capture approach in adolescent idiopathic scoliosis. Gait Posture. (2016) 44:231–37. 10.1016/j.gaitpost.2015.12.036 27004664

[3] Mao S Sun X Shi B Qiu Y Qian B Cheng JCY. Association between braced curve behavior by pubertal growth peak and bracing effectiveness in female idiopathic scoliosis: a longitudinal cohort study. BMC Musculoskelet Disord. (2018) 19:88. 10.1186/s12891-018-1987-9 29580223 PMC5870088

[4] Abreu N Dias I Cascais M Luz A Moleiro P. What are the most frequent diagnoses in adolescence? The reality of an adolescent medicine clinic. Einstein (Sao Paulo). (2018) 16:O4225. 10.1590/S1679-45082018AO4225 29972440 PMC6019239

[5] Tones M Moss N Polly DWJ. A review of quality of life and psychosocial issues in scoliosis. Spine (Phila Pa 1976). (2006) 31:3027–38. 10.1097/01.brs.0000249555.87601.fc 17173000

[6] Cheng JC Castelein RM Chu WC Danielsson AJ Dobbs MB Grivas TB Adolescent idiopathic scoliosis. Nat Rev Dis Primers. (2015) 1:15030. 10.1038/nrdp.2015.30 27188385

[7] Gur G Ayhan C Yakut Y. The effectiveness of core stabilization exercise in adolescent idiopathic scoliosis: a randomized controlled trial. Prosthet Orthot Int. (2017) 41:303–10. 10.1177/0309364616664151 27625122

[8] Herman R Mixon J Fisher A Maulucci R Stuyck J. Idiopathic scoliosis and the central nervous system: a motor control problem. The Harrington lecture, 1983. Scoliosis Research Society. Spine (Phila Pa 1976). (1985) 10:1–14. 10.1097/00007632-198501000-00001 3885413

[9] Akuthota V Nadler SF. Core strengthening. Arch Phys Med Rehabil. (2004) 85:S86–92. 10.1053/j.apmr.2003.12.005 15034861

[10] Chan YL Cheng JC Guo X King AD Griffith JF Metreweli C. MRI evaluation of multifidus muscles in adolescent idiopathic scoliosis. Pediatr Radiol. (1999) 29:360–63. 10.1007/s002470050607 10382215

[11] Kim H Lee C Yeom JS Lee JH Cho JH Shin SI Asymmetry of the cross-sectional area of paravertebral and psoas muscle in patients with degenerative scoliosis. Eur Spine J. (2013) 22:1332–38. 10.1007/s00586-013-2740-6 23515711 PMC3676542

[12] Mannion AF Meier M Grob D Muntener M. Paraspinal muscle fibre type alterations associated with scoliosis: an old problem revisited with new evidence. Eur Spine J. (1998) 7:289–93. 10.1007/s005860050077 9765036 PMC3611266

[13] Weiss HR. Imbalance of electromyographic activity and physical rehabilitation of patients with idiopathic scoliosis. Eur Spine J. (1993) 1:240–43. 10.1007/BF00298367 20054925

[14] Zoabli G Mathieu PA Aubin C. Back muscles biometry in adolescent idiopathic scoliosis. Spine J. (2007) 7:338–44. 10.1016/j.spinee.2006.04.001 17482118

[15] Wenxia Z Yuelong L Zhou Z Guoqing J Huanjie H Guifang Z The efficacy of combined physiotherapeutic scoliosis-specific exercises and manual therapy in adolescent idiopathic scoliosis. BMC Musculoskelet Disord. (2024) 25:874. 10.1186/s12891-024-07974-1 39482645 PMC11526564

[16] Weinstein SL Dolan LA Wright JG Dobbs MB. Effects of bracing in adolescents with idiopathic scoliosis. N Engl J Med. (2013) 369:1512–21. 10.1056/NEJMoa1307337 24047455 PMC3913566

[17] Lenke LG Betz RR Harms J Bridwell KH Clements DH Lowe TG Adolescent idiopathic scoliosis: a new classification to determine extent of spinal arthrodesis. J Bone Joint Surg Am. (2001) 83:1169–81. 10.2106/00004623-200108000-00006 11507125

[18] Ayhan C Unal E Yakut Y. Core stabilisation reduces compensatory movement patterns in patients with injury to the arm: a randomized controlled trial. Clin Rehabil. (2014) 28:36–47. 10.1177/0269215513492443 23823711

[19] Hides JA Boughen CL Stanton WR Strudwick MW Wilson SJ. A magnetic resonance imaging investigation of the transversus abdominis muscle during drawing-in of the abdominal wall in elite Australian football league players with and without low back pain. J Orthop Sports Phys Ther. (2010) 40:4–10. 10.2519/jospt.2010.3177 20044702

[20] Koumantakis GA Watson PJ Oldham JA. Supplementation of general endurance exercise with stabilisation training versus general exercise only. Physiological and functional outcomes of a randomised controlled trial of patients with recurrent low back pain. Clin Biomech (Bristol). (2005) 20:474–82. 10.1016/j.clinbiomech.2004.12.006 15836934

[21] Alves De Araujo ME Bezerra Da Silva E Bragade Mello D Cader SA Shiguemi Inoue Salgado A Dantas EHM. The effectiveness of the Pilates method: reducing the degree of non-structural scoliosis, and improving flexibility and pain in female college students. J Bodyw Mov Ther. (2012) 16:191–98. 10.1016/j.jbmt.2011.04.002 22464116

[22] Park J Jeon H Park H. Effects of the Schroth exercise on idiopathic scoliosis: a meta-analysis. Eur J Phys Rehabil Med. (2018) 54:440–49. 10.23736/S1973-9087.17.04461-6 28976171

[23] Berdishevsky H Lebel VA Bettany-Saltikov J Rigo M Lebel A Hennes A Physiotherapy scoliosis-specific exercises - a comprehensive review of seven major schools. Scoliosis Spinal Disord. (2016) 11:20. 10.1186/s13013-016-0076-9 27525315 PMC4973373

[24] Khaledi A Minoonejad H Akoochakian M Gheitasi M. Core stabilization exercises vs. Schroth’s three dimensional exercises to treat adolescent idiopathic scoliosis: a systematic review. Iran J Public Health. (2024) 53:81–92. 10.18502/ijph.v53i1.14685 38694867 PMC11058387

[25] Kocaman H Bek N Kaya MH Buyukturan B Yetis M Buyukturan O. The effectiveness of two different exercise approaches in adolescent idiopathic scoliosis: a single-blind, randomized-controlled trial. PLoS One. (2021) 16:e249492. 10.1371/journal.pone.0249492 33857180 PMC8049223

[26] Negrini S Donzelli S Aulisa AG Czaprowski D Schreiber S de Mauroy JC 2016 SOSORT guidelines: orthopaedic and rehabilitation treatment of idiopathic scoliosis during growth. Scoliosis Spinal Disord. (2018) 13:3. 10.1186/s13013-017-0145-8 29435499 PMC5795289

[27] Dimitrijevic V Raskovic B Popovic M Viduka D Nikolic S Drid P Treatment of idiopathic scoliosis with conservative methods based on exercises: a systematic review and meta-analysis. Front Sports Act Living. (2024) 6:1492241. 10.3389/fspor.2024.1492241 39763485 PMC11700739

[28] Dimitrijevic V Scepanovic T Jevtic N Raskovic B Milankov V Milosevic Z Application of the Schroth method in the treatment of idiopathic scoliosis: a systematic review and meta-analysis. Int J Environ Res Public Health. (2022) 19:16730. 10.3390/ijerph192416730 36554613 PMC9779560

[29] Dimitrijevic V Viduka D Scepanovic T Maksimovic N Giustino V Bianco A Effects of Schroth method and core stabilization exercises on idiopathic scoliosis: a systematic review and meta-analysis. Eur Spine J. (2022) 31:3500–11. 10.1007/s00586-022-07407-4 36229615

[30] Mohamed RA Yousef AM. Impact of Schroth three-dimensional vs. Proprioceptive neuromuscular facilitation techniques in adolescent idiopathic scoliosis: a randomized controlled study. Eur Rev Med Pharmacol Sci. (2021) 25:7717–25. 10.26355/eurrev_202112_27618 34982433

[31] Kuru T Yeldan I Dereli EE Ozdincler AR Dikici F Colak I. The efficacy of three-dimensional schroth exercises in adolescent idiopathic scoliosis: a randomised controlled clinical trial. Clin Rehabil. (2016) 30:181–90. 10.1177/0269215515575745 25780260

[32] Dimitrijevic V Raskovic B Popovic MP Viduka D Nikolic S Jevtic N Treatment of adolescent idiopathic scoliosis with the conservative Schroth method: a randomized controlled trial. Healthcare (Basel). (2025) 13:688. 10.3390/healthcare13060688 40150538 PMC11942212

[33] Jiang Y Peng H Song Y Huang L Chen H Li P Evaluating exercise therapies in adolescent idiopathic scoliosis: a systematic review with Bayesian network meta-analysis. PeerJ. (2025) 13:e19175. 10.7717/peerj.19175 40183057 PMC11967429

[34] Chen Y Zhang Z Zhu Q. The effect of an exercise intervention on adolescent idiopathic scoliosis: a network meta-analysis. J Orthop Surg Res. (2023) 18:655. 10.1186/s13018-023-04137-1 37667353 PMC10476432

[35] Otman S Kose N Yakut Y. The efficacy of Schroth's 3-dimensional exercise therapy in the treatment of adolescent idiopathic scoliosis in Turkey. Neurosciences (Riyadh). (2005) 10:277–83. 22473139

[36] Baik S Kim S Lee J. A scoping review of the different types of exercise programs proposed for the improvement of postural balance in adolescents with idiopathic scoliosis. J Back Musculoskelet Rehabil. (2023) 36:1261–72. 10.3233/BMR-220391 37482978

[37] Schreiber S Parent EC Khodayari Moez E Hedden DM Hill DL Moreau M Schroth physiotherapeutic scoliosis-specific exercises added to the standard of care lead to better cobb angle outcomes in adolescents with idiopathic scoliosis - an assessor and statistician blinded randomized controlled trial. PLoS One. (2016) 11:e168746. 10.1371/journal.pone.0168746 28033399 PMC5198985

[38] Burger M Coetzee W du Plessis LZ Geldenhuys L Joubert F Myburgh E The effectiveness of Schroth exercises in adolescents with idiopathic scoliosis: a systematic review and meta-analysis. S Afr J Physiother. (2019) 75:904. 10.4102/sajp.v75i1.904 31206094 PMC6556933

[39] Fang M Huang X Wang W Li Y Xiang G Yan G The efficacy of Schroth exercises combined with the Cheneau brace for the treatment of adolescent idiopathic scoliosis: a retrospective controlled study. Disabil Rehabil. (2022) 44:5060–68. 10.1080/09638288.2021.1922521 33984249

[40] Wang Z Zhu W Li G Guo X. Comparative efficacy of six types of scoliosis-specific exercises on adolescent idiopathic scoliosis: a systematic review and network meta-analysis. BMC Musculoskelet Disord. (2024) 25:1070. 10.1186/s12891-024-08223-1 39725973 PMC11670383

[41] Kucuk E Oten E Coskun G. Effects of spinal mobilisation in adolescent idiopathic scoliosis: a randomised controlled trial. J Paediatr Child Health. (2024) 60:660–68. 10.1111/jpc.16650 39152722

[42] Ong RYL Thazhakkattu Vasu D Jun LK Yuet NJ Isaac Fernandez M Selvakumar K Effectiveness of dynamic neuromuscular stabilization approach in lumbopelvic stability and gait parameters in individuals with idiopathic scoliosis: a randomized controlled trial. Medicine (Baltimore). (2025) 104:e41905. 10.1097/MD.0000000000041905 40128063 PMC11936624

